# Using Large Institutional or National Databases to Evaluate Prostate Cancer Outcomes and Patterns of Care: Possibilities and Limitations

**DOI:** 10.1100/tsw.2011.19

**Published:** 2011-01-18

**Authors:** Daniel Su, Thomas L. Jang

**Affiliations:** Division of Urology, Robert Wood Johnson Medical School, University of Medicine and Dentistry of New Jersey and The Cancer Institute of New Jersey, New Brunswick, USA

**Keywords:** prostate cancer, database, outcomes research, health-related quality of life

## Abstract

Prostate cancer is the most common non–skin-related cancer in men. With advances in technology, the care and treatment for men with this disease continues to become more complex. Large databases offer researchers a unique opportunity to conduct prostate cancer research in various areas, and provide important information that helps patients and providers determine prognosis after treatment. Furthermore, the studies using these databases may provide information on how side effects from various treatments can affect one's quality of life. Finally, information from these datasets can help to identify factors that determine why patients receive the treatments they do. Despite this, these databases are not without limitations. In this review, we discuss various available, national, multicenter, and institutional databases in the context of prostate cancer research, citing numerous important studies that have impacted on our understanding of prostate cancer outcomes.

## INTRODUCTION

Prostate cancer is the most common noncutaneous cancer in men, accounting for an estimated 217,000 new cases and over 32,000 deaths estimated for 2010[1]. While large national population databases have existed since the 1970s, it is due to the recent expansion in data processing capabilities that they are increasingly being used in epidemiological and outcomes studies. Large institutional databases also serve as a major source of data for important research that has advanced our understanding of prostate cancer outcomes.

Collectively, these datasets offer unique opportunities to study prostate cancer in several areas: comparative effectiveness of various treatment modalities in the absence of randomized studies, patterns of care, disparities in cancer care, health-related quality of life, quality of cancer care, and longitudinal outcomes. In fact, many major studies that have dramatically altered the care that is provided for prostate cancer patients have been published in recent years based on the use of large national and/or institutional databases. However, these databases are not without limitations and interpretation of outcomes data must be done critically when applying results to patient care.

This review aims to provide a general overview and applicability of the various national, multicenter, and large institutional databases available for prostate cancer research, to illustrate the types of studies that can be conducted using these data, and to discuss inherent limitations associated with their use. Certainly, many studies pertaining to prostate cancer outcomes have impacted patient care significantly and warrant mention. However, within the scope of this review, we are able to discuss only a select few, mainly to illustrate the wide variety of applications in which these databases can be used.

## OVERVIEW OF POPULATION-BASED DATASETS FOR PROSTATE CANCER RESEARCH

Population-based investigations using the various available national databases contribute significantly to the prostate cancer literature. These databases contain a large amount of information that includes tumor characteristics, sociodemographic factors, initial treatment, adjuvant or salvage treatments, data on cancer recurrence and patient survival, treatment-related complications that may impact quality of life, and costs of cancer care. Due to the population-based nature of these databases that capture a significant proportion of the national population, these datasets are able to generate large sample sizes that provide extraordinary statistical power. As the data are entered into these registries without influence from the care provider or referral center, national databases are able to avoid referral biases that may be seen in institutional datasets, thus being more reflective of the general population than data reported by tertiary care centers. Other potential advantages include the ability to track care for patients longitudinally over the course of cancer diagnosis, treatment, and follow-up, and across multiple provider settings, such as outpatient, inpatient, and hospice settings; and the capacity to evaluate and assess the quality of cancer care, disparities in heath care, and health care costs. Finally, in the absence of randomized clinical trials comparing the efficacy of various therapeutic modalities to treat prostate cancer, well-controlled studies using large population-based data can provide reasonable estimates of outcomes.

National population-based databases to be discussed include the Surveillance, Epidemiology, and End Results (SEER) program (and the Prostate Cancer Outcomes Study), the SEER-Medicare linked database, the Nationwide Inpatient Sample (NIS), and the National Cancer Database (NCDB).

## NATIONAL POPULATION-BASED DATABASES FOR PROSTATE CANCER RESEARCH

### The Surveillance, Epidemiology, and End Results Program (SEER)

The SEER cancer registry was the first national system for cancer surveillance and has been identified by the Institute of Medicine as one of the few population-based datasets available for the analysis of cancer-related care[2]. Established as one of the first steps in President Nixon's National Cancer Act of 1971, the SEER program aims “to provide information on cancer statistics to help reduce the burden of this disease on the U.S. population.” Data collection began on January 1, 1973 in the states of Connecticut, Iowa, Hawaii, New Mexico, Utah, and the metropolitan areas of Detroit and Oakland/San Francisco. Over the years, additional areas were added to the SEER program, including California, the metropolitan area of Atlanta and rural Georgia, the Seattle-Puget sound area, New Jersey, Louisiana, Kentucky, Arizona, and the Alaska native tumor registry. To date, SEER covers approximately 26% of the U.S. population, and includes 23% of U.S. African Americans, 40% of U.S. Hispanics, 42% of U.S. American Indians and Alaskan Natives, 53% of U.S. Asians, and 70% of U.S. Hawaiian/Pacific Islanders[3].

The SEER data are considered extremely valid with all of its registries holding the highest level of data quality certification as determined by the North American Association of Central Cancer Registries[4]. Completeness of case ascertainment is 98%. Data quality is rigorously evaluated annually in studies conducted in SEER regions[5]. SEER quality control and improvement activities include casefinding audits and reliability studies conducted in even-numbered calendar years and training programs for SEER registry personnel conducted in odd-numbered calendar years[6].

Clinical researchers using the SEER data should have a detailed working knowledge of the SEER data structure. The SEER program provides detailed information on a large number of individual cancer cases that includes, among other variables, year of diagnosis, state and county of residence at the time of diagnosis, demographic factors (such as age, gender, race, ethnicity and marital status) at diagnosis, detailed information on tumor characteristics, information on initial treatment, vital status at the end of follow-up, survival time, and the cause of death if deceased. However, SEER does not capture information on how the initial cancer was detected, nor does it capture data on the use of prostate cancer screening. Furthermore, SEER does not include information about patient comorbidities, treatment provided more than 4 months after diagnosis, or information about long-term disease status[5].

A thorough understanding of how variables in SEER are coded and how tumors are staged is critical in the proper design of a study using SEER data and in their interpretation. Coding and staging in SEER have evolved since its inception and differ significantly for cases diagnosed prior to 1988, cases from 1988 to 2003, and cases after 2004. The original SEER extent of disease (EOD) staging manuals and SEER program code manuals, as well as all subsequent revisions, are available at: http://seer.cancer.gov/tools/codingmanuals/historical.html. Various manuals may need to be carefully reviewed during study design depending on the research topic being addressed. For example, for years prior to 1998, stage was determined according to information obtained within the first 2 months following diagnosis. Since treatment often occurred after 2 months following diagnosis, only data on clinical stage (as opposed to pathologic stage) are available for many men with prostate cancer diagnosed before 1998. For cases diagnosed in 1998 to 2003, EOD codes were based on data available within the first 4 months following initial diagnosis in the absence of disease progression or through completion of initial treatment, whichever was longer. During this period, definitions of initial treatment also changed with time. Furthermore, in cases diagnosed in 1998 to 2003, pathologic tumor stage for prostate cancer was available and was based on EOD codes for pathologic extension[7]. Beginning with cases diagnosed in 2004, the SEER program coding and staging manual replaced EOD with Collaborative Stage (CS). As a result, pathologic tumor stage is now categorized according to CS Site-Specific Factor 3 CS Extension Code. Furthermore, clinical information previously unavailable, such as detailed pretreatment PSA information, surgical margin status, and primary and secondary Gleason scores are now captured in SEER cases diagnosed in 2004 and later[8].

Accessing SEER research data is free and can be done by signing a research data agreement that is required to access these data. The data files are available to download via an Internet connection or through disks shipped directly to researchers. Detailed information on accessing SEER datasets and tools is available at: http://seer.cancer.gov/resources/.

Use of SEER data is limited by the following: (1) lack of information on patient comorbidities; (2) lack of information on treatment-related complications; (3) lack of reliable measures of outcome for prostate cancer progression; (4) lack of central histology review; (5) lack of information on all initial treatments (i.e., if more than one surgery comprised initial treatment, only the most extensive surgery is captured); and (6) lack of information on chemotherapy. However, for SEER patients 65 years of age or older who are also Medicare beneficiaries, information on other treatments and chemotherapy not captured in SEER can be obtained from billing codes in the SEER-Medicare linked database, which is discussed below[7]. Furthermore, complications following prostate cancer treatments and assessing comorbidities is possible using Medicare claims data[9].

## SEER-Medicare Linked Database

The unique ability to link data from the SEER program to Medicare claims provides an additional ability to track patients across multiple providers, treatment settings, and disease states. Medicare is the primary health insurance for 97% of the U.S. population 65 years of age and older. Medicare Part A includes inpatient care, skilled nursing facilities, home health, and hospice care. Medicare Part B includes physician services, outpatient care, durable medical equipment, and home health. All Medicare participants receive Part A and 95% subscribe to Part B[10]. Medicare beneficiary information, including demographics and entitlement, is maintained in a master database known as the Enrollment Database (EDB).

Linkage between SEER and Medicare is a collaborative effort of the National Cancer Institute (NCI), the SEER program, and the Center for Medicare and Medicaid Services (CMS). It is based on an algorithm that matches the Social Security number, name, gender, and date of birth of all entries in SEER and EDB[5]. The linkage was first completed in 1991 and has been updated in 1995, 1999, 2003, 2006, and 2009. For each of the linkages, 93% of persons aged 65 years and older in the SEER files were successfully matched to the Medicare enrollment file. Linkages are done every 2 years. There is an approximate 2-year lag in data reporting in the SEER program. Medicare reporting has minimal lag[5].

Using data from the SEER-Medicare linked database, researchers are able to track prostate cancer patients from cancer diagnoses, to treatment, recurrence, or death, independent of care provider or location (outpatient, inpatient, rehabilitation, hospice). The linkage of these two complimentary, large, data sources (SEER program and Medicare claims) offers prostate cancer researchers the ability to assess patterns of care, quality of care, costs of care, and long-term outcomes. For example, the SEER database provides detailed information on diagnosis, stage, tumor characteristics, and cause of death, which is not found in Medicare claims. Data from Medicare files provide a long-term view of a patient’s cancer history, reporting information on the patient’s comorbidities and care before diagnosis, during treatment, during subsequent recurrence, and during any potential adjuvant/salvage treatments and/or chemotherapy. While SEER reports only the most invasive treatment, Medicare data capture all procedures related to a patient’s cancer care. Lastly, Medicare data provide a control population that is very useful in outcomes research. Medicare beneficiaries without cancer who live in SEER areas can be used as a control group in certain studies. A total of 262,534 cases of prostate cancer are captured in the SEER-Medicare files, from 1986 to 2005[11].

Given the large number of cases captured in SEER and the amount of Medicare claims associated with each case, the SEER-Medicare data are stored separately in the following different files: the Patient Entitlement and Diagnosis Summary File (PEDSF), the Medicare analysis and procedure file (MEDPAR), the National Claims History (NCH), hospital outpatient files, hospice/home health files, and the Summarized Denominator File for Non-Cancer Cases (SUMDENOM). The SEER data incorporated in the SEER-Medicare files are in a customized file (PEDSF), which contains one record per person for individuals in the SEER data who have been matched with Medicare enrollment records. Some of the data reported in PEDSF include the following: each person’s month and year of birth, date of death (if applicable), race, sex, county of residence, reason for Medicare entitlement, health maintenance organization (HMO) enrollment, median household economic and education status for the census tract or zip code where the person resides, and clinical information (such as tumor grade, stage, and histology, among others) for up to 10 diagnosed cancer cases. Medicare data in the SEER-Medicare files include claims from inpatient hospitalizations and procedures (MEDPAR files), hospital outpatient files, physician/supplier data (NCH files), and hospice/home health files. All Medicare files have data on age, race, sex, date of birth, date(s) of service, diagnostic codes, procedure codes, and reimbursements[12]. An excellent collection of review articles providing an overview of the SEER-Medicare data and their use in studying radiation therapy, cancer surgery, chemotherapy, and complications of cancer treatment, and their use in assessing comorbidities, has been published[5,13,14,15,16,17,18,19].

SEER-Medicare data are not public use data files. Researchers are required to submit a research proposal and obtain approval in order to obtain the data, to ensure the confidentiality of the patients and providers in SEER areas. Representatives from the NCI and SEER review each proposal. The review and approval process takes approximately 4–6 weeks from receipt of proposals. The cost of acquiring the SEER-Medicare linked data files is dependent on which data files are requested, the number of years of data files requested, and the number of primary cancer sites requested[11].

Prostate cancer studies using the SEER-Medicare database are subject to certain limitations. Medicare claims are created for reimbursement purposes, not research. Thus, information regarding the rationale for a procedure or test and its outcomes are unknown[5]. Because assessment of patient comorbidities and complications is based on administrative claims, it may be difficult to differentiate whether secondary diagnoses are comorbidities or complications. Administrative claims are subject to inaccurate coding as well as variability in coding practices among physicians, hospitals, and coders. Furthermore, complications that may not require a corrective procedure (i.e., artificial urinary sphincter for urinary incontinence or insertion of a penile prosthesis for erectile dysfunction) may be potentially under-reported, since reimbursement for physicians is driven by procedures. Also, many patients may not seek treatment for complications they experience and detailed information, such as patient-reported health-related quality of life data, serial PSA measurements, and specific clinical disease information, are not available. Another major limitation of SEER-Medicare data is the lack of claims information for HMO enrollees, as HMOs have not been required by the CMS to submit claims for services received by their Medicare enrollees. Up to December 2001, almost 14% of the nationwide Medicare population was HMO enrollees. Finally, prostate cancer studies using the SEER-Medicare data provide limited generalizability and insight on men <65 years of age diagnosed with prostate cancer.

Despite its limitations, studies using the SEER-Medicare linked database have contributed significantly to our understanding of prostate cancer treatments, outcomes, and health policy. It is beyond the scope of this review to detail all such studies, however, a few key examples that highlight the applicability and research potential of this database include the following:

1. Moderate reductions in Medicare reimbursement for androgen deprivation therapy (ADT) for prostate cancer starting in 2004 were associated with decline in ADT use, particularly among men for whom the benefits of such therapy were unclear, suggesting that reductions in reimbursement of treatments with no clear benefit may influence the delivery of care in a potentially beneficial way[20].
2. A significant proportion of men (41%) with clinically localized prostate cancer who did not receive definitive local therapy (i.e., surgery or radiation) were treated with primary androgen deprivation therapy (PADT) despite no difference in survival in the majority of elderly men who received PADT when compared to conservative management[21].
3. ADT use in this population increases the risks of cardiovascular morbidity[22], fracture[23], and incident diabetes among elderly men with prostate cancer[24].
4. Racial differences in mortality exist according to treatment received. For example, African American men had inferior survival when compared to white American men irrespective of whether they were treated with surgery, radiation, or nonaggressive methods[25].
5. Significant geographic variation exists nationwide on the surgery, radiation, and watchful-waiting treatment rates. Nonclinical factors, such as ethnicity and income, were associated with watchful waiting vs. surgery or radiation in men with early-stage prostate cancer[26].
6. The type of specialist seen by American men with clinically localized prostate cancer is strongly associated with ultimate treatment received. For example, younger Medicare beneficiaries who saw only a urologist more frequently received radical prostatectomy. In contrast, Medicare beneficiaries, irrespective of age, who also saw a radiation oncologist more often received radiation therapy[27].
7. Men undergoing radical prostatectomy have significantly reduced rates of postoperative and late urinary complications if the procedure is performed by a high-volume surgeon and/or in a high-volume hospital[28].
8. Men undergoing minimally invasive radical prostatectomy (MIRP) when compared to those undergoing open radical prostatectomy experienced shorter hospital stays, fewer respiratory complications and strictures, and lower rates of blood transfusions, but had higher rates of genitourinary complications, urinary incontinence, and erectile dysfunction[29].

## The Prostate Cancer Outcomes Study (PCOS)

The PCOS was initiated by the NCI in 1994 to investigate variations in initial treatment of prostate cancer and health-related quality of life (HRQOL) outcomes on a large scale. Detailed quality of life data were collected from men in six SEER registries who were diagnosed with prostate cancer from 1994 to 1995. A total of 5,672 men were eligible for the PCOS and 3,533 men participated. Advantages of this dataset include the maturity of follow-up for patients in the study and detailed HRQOL outcomes not available in any other population-based dataset. However, findings from this population may be limited by the evolving technology for prostate cancer care[30].

One key study using data from the PCOS showed that men with a history of a heart attack, who were unmarried, impotent, or had poor pretreatment bladder control, and who lived in certain geographic areas, were more likely to undergo conservative management. In this study, men who were 60 years of age or older and African American underwent aggressive treatment less often than white or Hispanic men[31]. Another study sampling 1,288 men 5 years after radical prostatectomy from the PCOS reported 14% of men with frequent urinary leakage or no urinary control, with 28% of men having preservation of erectile function. Sildenafil was the most commonly used erectile aid and its use was reported to help “somewhat” or “a lot” in 45% of users.

## Nationwide Inpatient Sample (NIS)

The NIS was developed as part of the Healthcare Cost and Utilization Project (HCUP). It is sponsored by the Agency for Healthcare Research and Quality. This database includes only hospital inpatient stays. However, it contains data from all payers, including the uninsured. The NIS comprises approximately 90% of all hospital discharges in the U.S., with data available from 1988 to 2008. The NIS data include primary and secondary diagnoses, primary and secondary procedures, admission and discharge status, patient demographics, expected payment source, total charges, length of stay, and hospital characteristics. The focus on cost and utilization from this database provides a unique opportunity to answer questions such as cost comparison between one form of prostate cancer treatment vs. another, variation in prostate cancer treatment based on location and type of hospital, and access to prostate cancer care and the utilization of health services by special populations[32].

Since the NIS captures data using administrative claims, it is subject to the same inherent limitations as the SEER-Medicare linked database previously mentioned. Other limitations specific to the NIS include the lack of long-term mortality data, lack of tumor-specific information (i.e., tumor stage), lack of data regarding neoadjuvant and/or adjuvant therapies, and variable and sometimes insufficient data regarding providers. Nonetheless, numerous studies on prostate cancer outcomes have been published using the NIS, the majority of them focusing on volume-outcome studies. For example, a cross-sectional study analyzing 61,039 men undergoing radical prostatectomy in 1,552 hospitals using 1998–2002 data from the NIS found that treatment at high- and moderate-volume hospitals was associated with lower odds of in-hospital mortality[33].

## National Cancer Database (NCDB)

The NCDB is a joint program of the Commission on Cancer (CoC) and the American Cancer Society (ACS). It provides a source of information on hospital patterns of cancer care and treatment outcomes thought to be representative of cancer care at the community level in the U.S. Its main purpose is to serve as a metric to enhance the quality of cancer care at the community level[34]. More than 1,400 Commission-accredited cancer programs in the U.S. are represented and approximately 70% of newly diagnosed cancers of all types are recorded. The NCDB started collecting data in 1989 and now contains more than 25 million records. Data elements are collected both on a longitudinal and cross-sectional basis. This database provides a resource to study and compare hospitals, create cancer survival reports, and report practice profiles, and, therefore, provides a unique resource for quality of care studies[35].

A major limitation of the NCDB is that the data are hospital-based, rather than population-based. Thus, interpretation of aggregate NCDB data and their national generalizability should be interpreted with caution. However, when SEER data from 1992 on 21,501 prostate cancers were compared with NCDB data on 107,690 prostate cancers, the following were noted: (1) SEER data were substantially more complete regarding Spanish or Hispanic heritage; (2) SEER prostate cancer historic staging data were less complete than NCDB data (i.e., 15.6% not recorded or unknown in SEER vs. 7.3% in NCDB); (3) data reporting for surgery vs. radiation was similar; (4) a greater proportion of SEER patients vs. NCDB patients received no cancer-directed surgery (48.7 vs. 42.8%), while similar proportions of SEER and NCDB prostate cancer patients were treated by radical prostatectomy (30.2 vs. 32.3%); (5) radiation therapy use was 8.1% greater among NCDB cases vs. SEER cases[36].

### Other National Databases

Other potential databases that can be used to assess prostate cancer outcomes include: (1) the National Program of Cancer Registries (NPCR), which are state-maintained, detailed cancer registries administered by the Centers for Disease Control[37]; (2) the National Surgical Quality Improvement Program (NSQIP), which can be used to compare various prostate cancer treatments[38]; and (3) databases based on national surveys, such as the National Health and Nutrition Examination Survey (NHANES)[39], the National Health Interview Survey (NHIS)[40], and the Medical Expenditure Panel Survey (MEPS)[41].

## MULTICENTER AND LARGE INSTITUTIONAL DATABASES FOR PROSTATE CANCER RESEARCH

Multicenter and institutional databases from tertiary care centers have also been used extensively in the study of prostate cancer care. While lacking the advantage of a large, representative sample population, data derived from tertiary care centers offer some unique advantages that national administrative databases lack. For example, far more detailed tumor characteristics may be available and captured, such as biopsy and pathologic Gleason scores, tumor laterality and focality, number of biopsy cores positive, total number of biopsy cores taken, prostate size, serial PSA measurements, margin status following radical prostatectomy, location of margins, and secondary treatments that are lacking in population-based datasets. As such, institutional databases are particularly well suited for cancer-specific outcomes research as they maintain detailed clinical information both pre- and post-treatment. These data are important for developing prognostic tools, such as nomograms, that can help patients and providers to more accurately determine prognosis following treatment[42]. Another advantage of large institutional databases is the ability to compare differences in treatment methods and outcomes in a controlled setting. For example, a study comparing clinical outcome for and cost of laparoscopic vs. robotic-assisted laparoscopic radical prostatectomies in the hands of a single surgeon at the same institution would not be possible using nationwide databases. Finally, data derived from tertiary care centers may potentially capture more specific patient- and provider-reported HRQOL outcomes that may be unavailable in population-based databases that utilize administration claims.

Multicenter databases to be summarized include the following: Carcinoma of the Prostate Strategic Urological Research Endeavor (CaPSURE), the Shared Equal Access Regional Cancer Hospital (SEARCH), and Center for Prostate Disease Research (CDPR).

## Carcinoma of the Prostate Strategic Urological Research Endeavor (CaPSURE)

CaPSURE was developed in 1995 as a disease registry of men with all stages of prostate cancer to describe national trends in disease management along with HRQOL and cancer outcomes data. CaPSURE is predominantly a community-based disease registry in which patients are enrolled currently from one of 31 urological practice sites (previously 40 sites), four of which are based at university centers and three of which are based at a Veterans Affairs (VA) medical center. The database is managed by the Urology Outcomes Research Group at the University of California-San Francisco (UCSF) and is funded by TAP Pharmaceutical Products[43].

One of the main strengths of the CaPSURE database is that validated HRQOL instruments are collected at baseline and longitudinally. This unique feature of CaPSURE allows investigators to analyze longitudinally the impact of various treatments (i.e., external beam radiation, brachytherapy, or radical prostatectomy) on HRQOL[43]. An excellent review of the CaPSURE database that summarizes its structure, organization, clinical data collected, and contributions to the prostate cancer literature has been published by Cooperberg and colleagues[43]. In addition to its significant contributions to our understanding of mens' HRQOL following various treatments for prostate cancer, CaPSURE has also described important information on practice patterns and oncologic outcomes. Select key findings reported by CaPSURE investigators include:

1. Among CaPSURE men diagnosed between 1989 and 1997, imaging studies such as computerized tomography and bone scans are overutilized in men with low-risk prostate cancer[44]. After 2001, rates of imaging are more strongly associated with disease risk (i.e., tumor stage, PSA, and Gleason score)[45].
2. The use of PADT over the last decade has increased significantly across all prostate cancer risk groups (i.e., low, intermediate, high) despite the lack of definitive evidence supporting its use in clinically localized prostate cancers, particularly in the low- to intermediate-risk setting[46].
3. More than half of men in CaPSURE on watchful waiting pursued secondary treatment within 5 years. Those who were younger or who had higher PSA levels at diagnosis were more likely to pursue secondary treatment, the most common of which was ADT[47].
4. Higher-staged prostate cancers, not surprisingly, were associated with higher costs when compared to lower-staged disease. The average cost of prostate cancer treatment in the first year following diagnosis was $6,375, and costs did not appear to differ significantly between patients receiving radical prostatectomy vs. external beam radiation[48].
5. A significant proportion of men with prostate cancer has their disease understaged and undergraded. For example, among 1,313 men in CaPSURE treated with radical prostatectomy, understaging (i.e., clinically localized, pathological stages T3 to T4 or N+) occurred in 24% of men and clinically significant undergrading (i.e., biopsy patterns 1 to 3 and pathological patterns 4 to 5) was found in 30% of specimens[49].
6. CaPSURE has also been used as a tool to externally validate well-established and popular prostate cancer prediction tools, such as the Partin Tables and the prostate cancer nomogram developed by Kattan and Scardino[50,51].

Despite the wide applicability of the CaPSURE database, certain limitations warrant mention. Despite data collection from broad practice settings, urology practices are over-represented and radiation practices are under-represented. Thus, studies using its data cannot be assumed to represent a statistically valid sample of prostate cancer practice patterns in the U.S., as the sites that enroll patients were not chosen at random. As an example, there appears to be an over-representation of white patients captured in CaPSURE when compared to national census data. Also, potential diagnostic and treatment bias may exist, as only diagnostic and therapeutic studies and interventions ordered by participating urologists are recorded. Finally, funding of the database is based on corporate sponsorship[43].

## Center for Prostate Disease Research (CDPR)

The Department of Defense CDPR Multi-Center National Prostate Cancer Database was initiated in 1994 as a single-center study to collect prospective and retrospective data on military health care beneficiaries. The program expanded to include 12 military sites in 1997–1998, but as of 2001, three have been eliminated, leaving nine contributing sites. Over 12,000 men have been enrolled since July 1999. Data elements include information on tumor stage, PSA level, prostate biopsy, treatment modality (i.e., radical prostatectomy, external beam radiation, hormone therapy, brachytherapy, cryotherapy, etc.), and radical prostatectomy pathology, among others[52,53].

A significant finding using data from the CDPR was the initial discovery that PSA levels are higher in African American men with newly diagnosed prostate cancer when compared to levels in white men, after controlling for age, tumor grade, and tumor stage[54]. The military-based registry is advantageous in that a high proportion of African American and Hispanic men are represented, thus allowing for studies of ethnicity and prostate cancer. However, the closed military health system biases against men who are indigent and of low socioeconomic status, as these men are poorly represented in CDPR. Furthermore, the ability to link tissue bank to clinical data using CDPR has allowed for biomarker studies (i.e., p53, p16, bcl-2, NKX3-1, among others) in radical prostatectomy patients[55,56,57].

## Shared Equal Access Regional Cancer Hospital (SEARCH)

The SEARCH database is a multicenter database, first reported in 2002, that represents combined radical prostatectomy data from four VA hospitals and one military hospital. Overlap among SEARCH, CPDR, and CaPSURE exists, as the San Diego Naval Hospital contributes to both CPDR and SEARCH, and the San Francisco VA contributes to both CaPSURE and SEARCH[58].

A PubMed search as of October 2010 using keywords “SEARCH database AND prostate cancer” reveals almost 50 publications attributable to the SEARCH database. A few recent and key findings include:

1. Men with diabetes mellitus and poorer glycemic control presented with biologically more aggressive prostate cancers when compared to those with better glycemic control[59].
2. Statin use was associated with a dose-dependent reduction in the risk of PSA recurrence after radical prostatectomy, after adjusting for multiple clinical and pathologic characteristics[60].
3. Obesity was associated with a greater risk of disease recurrence following radical prostatectomy among both African American and white men, and independently was associated with more aggressive cancers, irrespective of age[61].
4. PSA doubling time (PSADT) is not calculable in a substantial proportion of men (about 35%) with PSA recurrence following radical prostatectomy and, therefore, is limited in its ability to risk stratify men with a biochemical recurrence after surgery. Men in whom PSADT was calculable represented a select, lower-risk cohort[62].
5. The use of PSA velocity >2 ng/ml/year was associated with a higher risk of relapse after radical prostatectomy, but its clinical utility may be limited to nonobese men[63].

Since SEARCH is a radical prostatectomy–based database, information is lacking on prostate cancer patients treated with other modalities. Furthermore, predominantly VA-based data ascertained from SEARCH may not accurately reflect the general population. However, the large proportion of African American men captured in SEARCH, similar to the CDPR database, makes this an ideal dataset to evaluate ethnicity and prostate cancer outcomes following surgery.

## LARGE INSTITUTIONAL DATABASES

Tertiary care centers, such as Memorial Sloan-Kettering Cancer Center (MSKCC) and Johns Hopkins, among others, maintain extensive and detailed databases for prostate cancer patients. Advantages of large institutional databases vs. national databases have been previously mentioned. Given more detailed tumor-specific information, these databases are better positioned to undertake studies such as the utility of PSA kinetics and outcomes, and to develop prognostic tools that help patients and providers predict outcomes following treatment. It is beyond the scope of this review to summarize and detail all large institutional databases that have made contributions in this field. However, a select few are outlined below.

PSA has greatly altered the way prostate cancer is screened, treated, and followed. The landmark study by Catalona and colleagues between 1989 and 1990 provided the basis for prostate cancer screening in the U.S. The authors measured serum PSA in 1,653 healthy men without a history of prostate cancer and performed transrectal prostate biopsies on those with a PSA level >4.0 μg/L. They found that between PSA measurement, digital rectal exams (DRE), and ultrasonography alone, PSA measurement had the lowest error rate in missing prostate cancer. PSA measurement plus digital rectal exam had the lowest error rate of the two-test combinations[64]. Based on the findings in this study, PSA became the standard of care in combination with DRE for prostate cancer screening.

PSA kinetics and parameters, such as velocity, doubling time, and percentage-free PSA, are now routinely used clinically as predictors of outcome and triggers for prostate biopsy. A key study conducted by Catalona and associates found that a cutoff value of 25% free PSA would detect 95% of cancers, while avoiding 20% of unnecessary biopsies. The study evaluated 773 men 50–75 years of age with PSA values 4–10 ng/L with pathologic diagnosis of BPH or prostate cancer, and found that percentage-free PSA may be used as a single trigger value for biopsy or to aid in patient risk assessment[65].

An elevated PSA after radical prostatectomy signifies biochemical recurrence. However, the time course to metastasis and death in this clinical state was undefined until after a landmark study addressing this topic was published by Pound and colleagues using the Johns Hopkins database. In their study, 1,997 men who underwent radical prostatectomy for localized disease were followed between 1982 and 1997. A detectable PSA level of 0.2 ng/mL or greater was considered biochemical recurrence. The median time to metastasis from the time of PSA elevation was 8 years. Once prostate cancer metastasized, the median time to death was 5 years. This study also showed that the time necessary for the PSA to double after surgery, or the PSADT, was one of the best predictors of developing metastasis[66].

Using Dr. Catalona's radical prostatectomy outcomes database, D'Amico and associates found that men who had a preoperative PSA velocity (PSAV) >2.0 ng/ml during the year before diagnosis were at a high risk of death from prostate cancer despite undergoing surgery[67]. Multiple studies have confirmed the association between PSAV and outcome. However, the utility of PSAV to provide predictive information beyond pretreatment PSA level alone has not yet been universally confirmed by other investigators[68,69].

Important prognostic tools using pretreatment variables have been developed using large institutional databases. A popular prognostic tool described by Partin and colleagues in 1997 combined data on 4,133 men from Johns Hopkins, Baylor/MSKCC, and the University of Michigan. This tool, now referred to as the Partin Tables, uses a combination of PSA level, clinical stage, and Gleason score to predict pathological stage at the time of radical prostatectomy for men with localized prostate cancer[70]. The prostate cancer nomogram developed by Drs. Kattan, Scardino, and colleagues from MSKCC has enabled providers and patients to more accurately determine outcome following radical prostatectomy. The nomogram was developed using data from almost 1,000 men treated by surgery for prostate cancer at MSKCC and Baylor College of Medicine. Initially described in 1998 to describe the 5-year probability of failure after radical prostatectomy, the nomogram has since been updated using data on almost 2,000 men to predict a patient's probability of remaining cancer free 10 years following treatment. Based on preoperative PSA, biopsy Gleason score, clinical stage, number of biopsy cores positive for cancer, and the total number of biopsy cores taken, an estimate of outcomes 10 years following treatment can now be predicted[42,71]. Nomograms using information from postoperative pathologic information have also been developed by the same MSKCC investigators[72,73]. Furthermore, nomograms to predict outcomes for prostate cancer patients treated with brachytherapy, three-dimensional conformal radiotherapy, and intensity-modulated radiotherapy have also been developed[74,75].

MSKCC investigators have also described the independent and important role of surgeon experience on outcomes after radical prostatectomy. In a study evaluating 4,629 men treated with radical prostatectomy, positive surgical margin rates ranged from 10 to 48% among 26 surgeons who each treated more than 10 patients, with higher-volume surgeons experiencing lower rates of positive surgical margins[76]. In a separate study using combined data from MSKCC, Baylor, the Cleveland Clinic, and Wayne State University, the influence of surgeon experience on cancer control after radical prostatectomy has been described. In this study, which included 7,765 men treated with radical prostatectomy, cancer control after radical prostatectomy improved as a surgeon's experience improved[77].

Finally, a comprehensive multicenter study involving nine university-affiliated hospitals that assessed quality of life and satisfaction with outcome among prostate cancer survivors was recently published. The investigators collected prospective HRQOL information using validated instruments, such as the expanded Prostate Cancer Index Composite (EPIC-26) and Service Satisfaction Scale for Cancer Care (SCA). Data were reported on 1,201 patients and 625 spouses before and after radical prostatectomy, brachytherapy, or external beam radiotherapy. Each prostate cancer treatment was associated with a distinct pattern of change in HRQOL domains related to urinary, sexual, bowel, and hormonal function. Furthermore, these changes influenced satisfaction with treatment outcomes among patients and their spouses or partners[78].

### Limitations of Large Institutional Databases

While large institutional datasets tend to offer more detailed information, they often lack the raw sample size of a nationwide database. Institutional databases tend to offer data on only a select segment of the general population, whether limited by geography, access to care, or pattern of care at a specific institution. This makes studies based on these types of databases less generalizable to the U.S. population as a whole. The fact that most institutions do not accept all insurance policies further limits the ability to study the underserved population. Referral bias is also often seen. For example, tertiary care centers tend to treat patients who are clinically complicated or who have advanced disease, and this is further complicated by differences in patterns of referral. For example, center A may receive heavy radiation treatment referrals for localized prostate cancer, whereas in center B, this group of patients is more often referred for surgery. The validity and quality of data with institutional databases should also be considered. Since most institutions are expert at few, but not all, procedures, e.g., minimally invasive prostatectomy, but not radical retropubic prostatectomy, it is often difficult to compare treatment outcomes within institutions. Some recent studies have overcome this limitation by combining data from multiple centers. Due to the limited number of surgeons at a single institution, studies for outcomes in relation to surgical volume are also challenging. Finally, access to care is often hard to assess since these databases tend to include a very low percentage of uninsured and/or medically indigent patients.

## CONCLUSIONS

With the advances in technology, the care and treatment for prostate cancer patients continues to become more complex. Due to the lack of randomized clinical trials comparing different treatment modalities, decision making for treatment becomes an issue that is patient- and provider-dependent. In the PSA era, more men are diagnosed with prostate cancer at an early stage, and this places further emphasis on treatment side effects that a patient may need to cope with life long. In this setting, outcome studies, particularly those focused on HRQOL, have become very important for our understanding of the disease and how it affects the patient. Furthermore, improved survival outcomes pertaining to treatment remain the goal. Data from population-based cancer registries or from large institutions that gather detailed information on prostate cancer patients have thus become a crucial resource for our understanding of this disease. When used appropriately, these datasets can be powerful tools that enable researchers to answer questions about prostate cancer that may not be feasible to answer otherwise.
